# Study of a Deep Convolution Network with Enhanced Region Proposal Network in the Detection of Cancerous Lung Tumors

**DOI:** 10.3390/bioengineering11050511

**Published:** 2024-05-19

**Authors:** Jiann-Der Lee, Yu-Tsung Hsu, Jong-Chih Chien

**Affiliations:** 1 Department of Electrical Engineering, Chang Gung University, Taoyuan 33302, Taiwan; 2 Department of Neurosurgery, Chang Gung Memorial Hospital at Linkou, Taoyuan 33305, Taiwan; 3 Department of Electrical Engineering, Ming Chi University of Technology, New Taipei City 24330, Taiwan; 4 Department of Information Communication, Kainan University, Taoyuan 33857, Taiwan

**Keywords:** deep learning, improved faster R-CNN, cancer

## Abstract

A deep convolution network that expands on the architecture of the faster R-CNN network is proposed. The expansion includes adapting unsupervised classification with multiple backbone networks to improve the Region Proposal Network in order to improve accuracy and sensitivity in detecting minute changes in images. The efficiency of the proposed architecture is investigated by applying it to the detection of cancerous lung tumors in CT (computed tomography) images. This investigation used a total of 888 images from the LUNA16 dataset, which contains CT images of both cancerous and non-cancerous tumors of various sizes. These images are divided into 80% and 20%, which are used for training and testing, respectively. The result of the investigation through the experiment is that the proposed deep-learning architecture could achieve an accuracy rate of 95.32%, a precision rate of 94.63%, a specificity of 94.84%, and a high sensitivity of 96.23% using the LUNA16 images. The result shows an improvement compared to a reported accuracy of 93.6% from a previous study using the same dataset.

## 1. Introduction

In recent years, artificial intelligence (AI) has become one of the fastest-growing fields in science and technology, and its explosive development allows it to take an increasingly important role in various fields, including but not limited to medical image processing. It can improve the results of image processing by being able to handle large amounts of data with very little human input. At the same time, the amount of data also grows explosively due to the development of information and medical technologies. These separate developments enabled each other to increase their respective rate of development in a mutually beneficial relationship. The developments of AI technology have brought new methods and ideas to the applications of processing medical images, making medical image diagnosis faster, more efficient, and more reliable. Therefore, the main purpose of this paper is to explore how to use AI technology to detect objects, specifically malignant lung nodules, in medical images and focus on how to perform deep learning and object detection on slice-combined images. Through this new method, we can better discover hidden information in medical imaging data and help physicians better understand and treat various diseases.

In this paper, a deep convolution neural network based on the structure of the faster R-CNN with an enhanced region proposal network (RPN) is proposed. This structure should capture the locations of the objects of interest more accurately and with better sensitivity than the original faster R-CNN network. So, in order to test this architecture, CT images of patients with cancerous tumors are used for this investigation, and the results will be compared with similar architectures without enhanced RPN. According to the GLOBOCAN data in 2020, a total of about 19.3 million cancer cases and about 10 million deaths caused by cancer were reported globally that year. Among these cancer cases, the three most common ones were breast cancer (2.26 million cases, accounting for 11.7%), lung cancer (2.21 million cases, accounting for 11.4%) and prostate cancer (1.41 million cases, accounting for 7.3%); in terms of mortality, the top three causes are lung cancer (1.79 million cases, accounting for 18%), liver cancer (830,000 cases, accounting for 8.3%) and gastric cancer (769,000 cases, accounting for 7.7%) [1]. It can be seen that the incidence and mortality of lung cancer have a significant impact on people.

Pulmonary nodules are representative lesions during the early stages of lung cancer, which are high-density lesions that exist in the human lungs. However, most of the time, the malignant nodules are discovered late, and the optimal windows for treatment are usually passed. This is also the reason why the mortality rates for lung cancer remain high worldwide [2]. Thus, regular lung testing is important.

In terms of current mainstream imaging technologies, low-dose computed tomography (LDCT) is currently the mainstream method of lung imaging and is also used as the diagnostic basis for radiologists to detect lung cancer. Most of the detection methods for pulmonary nodules rely on the judgment of radiologists based on their years of clinical experience. However, CT images usually consist of hundreds of images. In addition, the number of lung cancer patients has been rising in recent years [3]. This has not only caused the radiology department diagnosticians to have to face more CT imaging data but also increased their workload and diagnostic pressure.

LUNA16 was originally a dataset for the 2016 Lung Nodule Detection Challenge (Lung Nodule Analysis) [4]. It aimed to promote the development of automated detection of lung nodules, increase the probability of early detection of lung cancer, and promote the field of computer vision in medical imaging. LUNA16 helped researchers develop automated lung nodule detection and classification methods. This dataset provides a large number of labeled pulmonary nodule images, as well as related clinical information and medical record data, and has become one of the most important datasets in the field of pulmonary nodule detection.

The LUNA16 dataset was compiled from another dataset that is used for lung image analysis—the LIDC-IDRI dataset, and these affected areas within the images were annotated by four professional researchers. In the end, a total of 888 sets of LDCT scans of anonymous patients were obtained, and for each set of scans, there may be multiple sets of labeled areas. The size of each set of slice images is 512 × 512 pixels. For each set of CT scans, the researchers only retained nodules that were greater than or equal to 3 mm and marked the location and size of each nodule. In addition, this dataset also provides negative sample images of non-pulmonary nodule areas, which are very similar in appearance to lung nodules. These labeled samples are helpful in identifying nodules and non-nodule areas during training, even though the number of positive and negative samples is unbalanced.

For the LUNA16 dataset, which contains uniform slice-type combined images, there are roughly two types of neural network choices that can be used for training: 2D and 3D. Generally speaking, whether it is accuracy or sensitivity, the performance of neural networks using 3D is often better than 2D, but 3D networks require larger amounts of parameters and consumptions of computational resources. So, how to strike a balance between 2D and 3D will be discussed later.

The purpose of this investigation, using the LUNA16 dataset, is to explore incremental improvement by tweaking the architecture for the Region Proposal Network. These tweaks include modifying the regression and classification layers and incorporating attention models into the classification layer, which should improve the performances in the detection of tumors and the reduction of false positives.

## 2. Related Works

At present, many pulmonary nodule detection methods are used in the LUNA16 dataset for training, and many of these, which are different types of neural networks, have achieved good performance. Chao et al. [5] proposed the ISODATA method, which uses the faster R-CNN as a detection model and uses the k-means grouping method to generate anchors suitable for labeling block sizes. The neural network classifiers used by Chao [5] contained both 2D and 3D, and the 3D CNN was used for comparison. However, in order to solve the problem of the imbalance in the number of positive and negative samples in the dataset, Chao [5] added the Focal loss function to the 3D CNN. Yuemeng Li et al. [6] used their proposed method, DeepSEED, in the region proposal network (RPN) of the faster R-CNN architecture in order to implement dynamic cross-entropy, which can solve the problem of imbalance between positive and negative samples and has shown to achieve a good performance. This network is called a lightweight attention mechanism model because the weight of each channel can be adaptively adjusted to increase the importance of the useful features. DeepSEED uses a technology similar to RPN to generate candidate regions of nodules and then passes these generated regions to the decoder for subsequent processing and classification. They reported a sensitivity of 0.920, and an average free-response ROC of 0.862 was achieved using the LUNA16 dataset. Zhao et al. [7] used ResNet as the encoder and U-Net as the decoder. The authors added some multi-scale feature extraction and attention mechanism blocks to certain residual blocks in the ResNet to make the network perform better. The characteristics of learning nodules were used to improve the performance of the sorting network, and a sensitivity of 0.935 was reported. Lu et al. [8] first used U-Net to segment the lungs, and the RPN network was used to generate ROI for the segmented blocks and squeeze-and-excitation to enhance important features and weaken unimportant ones. The influence of these features resulted in an overall sensitivity of 0.937 in their report. Shuvo [9] used Res-U-Net when segmenting lung images. Res-U-Net integrates the concept of residual connection into U-Net and adds the residual blocks together to improve the model’s performance and stability and avoid the problem of gradient disappearance in U-Net. Shuvo [9] used YOLO-v5 to localize the locations of nodules and a vision transformer to classify the nodules and reported an accuracy of 93.57 and a sensitivity of 93.10, respectively. The 3D convolutional neural network proposed by Chen et al. [10] simulated adding image enhancement noise to the CT images during the training process in order to help train the model, similar to the effect of image enhancement used in image augmentation, thereby improving the accuracy of pulmonary 10identification. Their reported performance has reached a sensitivity of 0.945. Yu et al. [11] proposed an improved 3D faster R-CNN architecture. The proposed network uses a densely connected network to achieve feature propagation and reduce the problem of gradient disappearance and reach a sensitivity of 0.952. Liu [12] proposed a detection method called STBi-YOLO, which consists of three parts: first, the input image is extracted through a convolution layer, and then multi-layer convolution and summation are performed through SPP-Net. Pooling, then feature fusion, is performed through the improved FPN network to obtain richer features and improve the detection accuracy of YOLO-v5, which is used for detection. The EIoU loss function was used for model training and optimization. They reported reaching an average of 0.936 in accuracy using the LUNA16 dataset.

In 2D + 3D methods, Ding et al. [13] used an architecture similar to faster R-CNN and used the VGG16 network as the feature extractor. Similar to the method being proposed in our research, they used a 2D feature extraction architecture and a 3D classifier. This is to reduce the generation of false positives and expand the amount of data through cropping and flipping in order to increase samples for training. The use of three-dimensional deep convolutional networks (DCNN) can comprehensively capture more contextual information. They reported achieving a sensitivity level of 0.946. Banu et al. [14] proposed an architecture called AWEU-Net, which is divided into two stages. The first stage uses the faster R-CNN architecture and uses ResNet for feature extraction to find the possible locations of all nodules. The second stage uses ResNet and U-Net as the encoder and decoder, respectively, and incorporates the PAWE/CAWE (position/channel attention-aware weight excitation) method. PAWE can grasp the relationship between feature context information, and CAWE can grasp the interdependence between channels in feature maps. By weighting that information into the local features, the two mechanisms can help the model better learn the ability to distinguish between positive and negative samples. The reported overall accuracy was 0.913, and the sensitivity was 0.916. Nguyen et al. [15] also used a hybrid faster R-CNN architecture similar to the method in our research and used the adaptive anchor box method to improve feature extraction. They generate anchors to capture the target position by corresponding feature sizes and use the ResNet model as the classifier. The generation of false positive targets was reduced and achieved high accuracy and specificity, which were 95.7% and 97.6%, respectively. Also, a sensitivity of 0.975 was achieved when the network that reduces false positive targets was not used.

## 3. Materials and Methods

**Figure 1:**
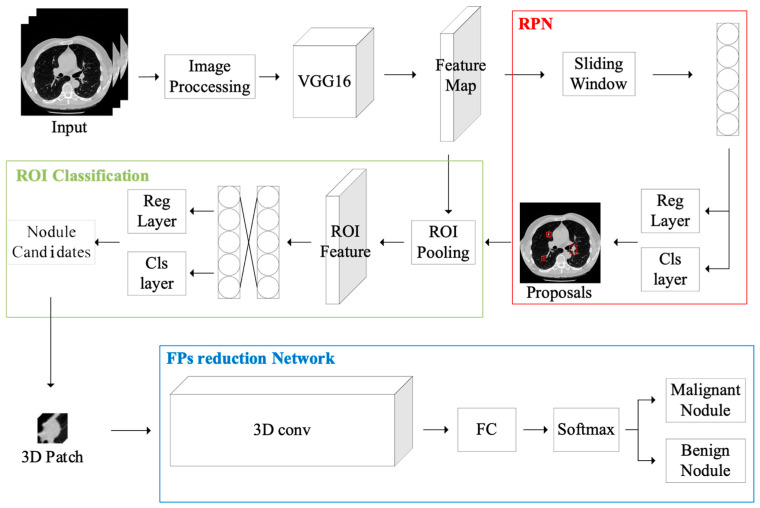
The system flowchart of this architecture, from preprocessing to region proposals to the classification of regions, then finally to the reduction of the positive positives before output.

Our investigation started with the faster R-CNN deep learning model [16] for the purpose of detecting pulmonary nodules in CT images. In order to enhance the model’s generalization capabilities, a series of image preprocessing steps were first applied to the LUNA16 dataset. These steps included lung segmentation, image scaling, normalization, and other operations. The processed images are then served as input to the faster R-CNN network, where feature maps are extracted and then fed to the proposed RPN network, which is supposed to improve the detection of candidate regions. The proposed regions are used for feature extraction, which are then classified and assessed as candidate boxes, resulting in the localization and sizing of possible nodules. For the classifier component, both 2D and 3D CNN neural networks were used for classification, and their performance differences were compared. Finally, attention models were integrated to enhance the identification of malignant and benign nodules. Figure 1 illustrates the system flowchart of the proposed system.

### 3.1. Preprocessing

**Figure 2:**
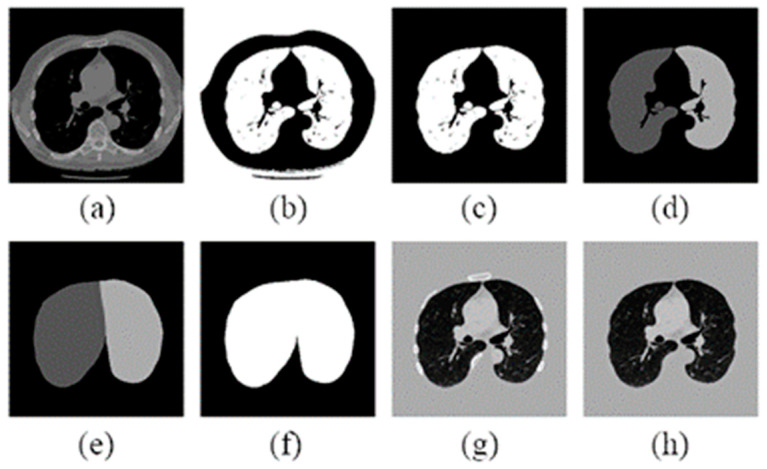
Segment the lung image after a series of preprocessing: (**a**) convert the source image into HU value; (**b**) binarize the image; (**c**) remove non-lung areas; (**d**) find the left and right distribution of the right lung; (**e**,**f**) expanded lung area; (**g**) HU converted to UINT8 after multiplication; and (**h**) bone part removed.

This investigation uses the preprocessing method proposed by Liao et al. [17] on the CT images. This method primarily employs image binarization to segment regions that may contain pulmonary nodules. Examples of the results after using this method are shown below in Figure 2. The preprocessing method uses the Hounsfield Unit. The Hounsfield unit (HU) serves as a standardized quantitative scale for describing the radiodensity of tissues. Every tissue has a specific HU value range [18], and this range remains consistent across different individuals and scanning equipment. Converting raw data into HU values allows for more convenient comparisons and analyses. Higher HU values generally correspond to denser tissues, while lower HU values represent sparser tissues, improving the distinction between lung nodules and other tissues for enhanced diagnostic accuracy.

To obtain an image containing only the lungs, a masking method is employed by multiplying the original images. When the image is input, different HU values yield distinct parts. In the (a) image in Figure 2, tissues with poor X-ray absorption, such as the lungs or air, appear black in the image. Subsequently, in the (b) image, the grayscale of the image is inverted and then binarized. The white area representing the shape of the lungs in the center is extracted, and the white region outside the body is filled in to obtain an image that exclusively includes the central lungs in the (c) image. In order to account for potential image flips during the CT scanning process, as shown in the (d) image, the distribution areas of the left and right lungs are determined based on the direction of the white area in the image. These areas are then expanded to cover the entirety of the lungs, ignoring potential areas where nodules might be attached to the lung walls. Finally, a black-and-white mask is generated, as seen in images (e) and (f). After obtaining the mask corresponding to the image, the image is converted from HU to UINT8 format, ensuring that the mask values fall within the [0, 255] grayscale range. The mask is then multiplied with the original image, resulting in the image in the middle, as shown in the (g) image. Occasionally, bright white areas caused by bones surrounding the lungs may appear in the outer ring. These are removed, as shown in the (h) image, using edge detection, resulting in the lung area.

### 3.2. Feature Extraction

When selecting a target detection method, various deep learning model architectures are available, including the R-CNN, the fast R-CNN, the faster R-CNN, and the mask R-CNN, among others. After careful evaluation, this study opted for the faster R-CNN as the deep learning model.

The faster R-CNN architecture, an enhanced version of the fast R-CNN, introduces a neural network architecture known as the region proposal network (RPN). RPN’s basic structure relies on a technique called selective search to generate candidate regions. This process involves dividing each image and drawing boundaries, calculating different blocks based on the color or texture of the boundary regions, and iteratively merging blocks with high similarity until all blocks are consolidated into one. These merged blocks constitute the candidate regions generated by selective search. While this method is effective, it occasionally exhibits drawbacks, such as an unstable number of candidate regions and difficulty handling overlapping targets. Faster R-CNN addresses these challenges by using convolutional layers to extract image features and employing an improved RPN network to generate candidate regions. The core principle is to identify the most likely location where the feature corresponds to the target. During the candidate region generation process, RPN generates multiple anchor boxes for each feature center position to specify potential object locations and sizes. For each anchor box, RPN outputs a binary classification score (target or background) and a set of offsets. The anchor box with the highest intersection over union (IOU) is adjusted to best fit the actual box’s position and size. These generated candidate regions are then input into the region of interest (ROI) network for feature extraction and scaling and ultimately classified by the final convolutional neural network (CNN) model.

The faster R-CNN architecture not only rectifies the limitations of the original fast R-CNN, such as the unstable number of candidate regions and challenges in handling overlapping targets, but also significantly improves the precision of target detection. Furthermore, faster R-CNN has the advantage of being easier to implement and compatible with various other deep learning frameworks.

**Figure 3:**
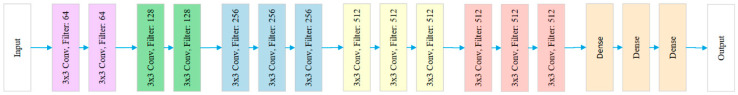
Architecture of VGG16, which is used for feature extraction and fine-tuning of images.

In the feature extraction process using faster R-CNN, we employed the VGG16 neural network, as illustrated in Figure 3. VGG16 is a model introduced by the Oxford Visual Geometry Group at the University of Oxford during the 2014 ImageNet competition [19]. This model, characterized by 13 convolutional layers and 3 fully connected layers, achieved an impressive accuracy rate of over 90% at that time. To facilitate feature extraction, we modified the last fully connected layer to contain two neurons, allowing us to capture the necessary features. These extracted features were subsequently fed into the 3D classifier.

### 3.3. RPN Network

As previously mentioned, the features extracted by VGG16 are initially directed to the region proposal network (RPN) to generate and propose candidate regions of interest, which are then passed to the ROI (region of interest) layer along with the original input features for subsequent processing.

The primary objective of the RPN network is to generate candidate regions that potentially contain the target object based on feature size. The following steps provide a concise overview of how RPN operates:

1. Feature extraction from input: initially, features are extracted from the input image using a convolutional neural network through multiple convolutional and pooling layers. These features are subsequently fed into both the RPN network and the ROI layers.
2. Sliding window: a sliding window with a fixed size is employed for sliding, with each window area being referred to as an anchor box. Each anchor box is designed with various scales and aspect ratios.
3. Anchor box regression: RPN predicts the offset for each anchor box to accurately adjust its position and size, making it more closely aligned with the target object.
4. Anchor box classification: additionally, RPN predicts whether each anchor box contains the target object, performing binary classification to distinguish between the target (positive sample) and background (negative sample) anchor boxes.
5. Region selection: based on the classification scores of the anchor boxes, RPN ranks the regions according to their predicted scores and selects the region most likely to contain the target as the candidate region. Typically, the top N regions are chosen based on score ranking.

In the process of generating an anchor frame from a feature, the offset is iteratively adjusted after an initial value is assigned to bring the frame closer to the actual size of the feature. When generating the anchor frame, a significant difference between the initial value and the feature size can result in a longer generation time. Conversely, if the initial value is closer to the feature size, it may increase the number of parameters but shorten the generation time. To address this, we employ a clustering approach to compare the performance of the k-means clustering algorithm with the fuzzy c-means clustering algorithms for our architecture.

In the k-means clustering, the process begins with an initial choice of ‘k’ value. K data points are randomly selected as the initial cluster centers. The distance between each data point and the individual cluster center is calculated, and each data point is assigned to the nearest cluster center. Subsequently, the individual cluster centers are updated sequentially and set to the average value of the data points within each cluster. This process of recalculating distances and updating cluster centers is repeated until the center values no longer change or meet the preset stopping conditions, thereby finalizing the clustering.

Fuzzy c-means is a clustering algorithm based on fuzzy theory. Compared with traditional clustering algorithms (such as the k-means algorithm), fuzzy c-means allow each data point to belong to multiple clustering centers, thus giving more flexible clustering results. Equation (1) is about the calculation method of making each data point correspond to different cluster center membership degrees in the fuzzy c-means algorithm:

(1) $$u_{i}={\left(\sum_{k=1}^{c}\left({\left(\frac{d_{\mathrm{ij}}}{d_{\mathrm{kj}}}\right)}^{\frac{2}{m-1}}\right)\right)}^{-1}$$

where ${\;u}_{i\;}$ is the corresponding membership degree,  c  is the number of clusters, m  is the fuzzy factor, and d is the distance between each data point corresponding to different cluster centers. The fuzzy factor in this experiment m is given as 2. If the fuzzy factor is too large, the clustering result will be unstable, and the position of the cluster center will be fuzzier, which will increase the uncertainty of training; if the fuzzy factor is too small, the clustering result will be unstable, such as if the degree is closer to 0 or 1, it would make the clustering results clearer, similar to the clustering results after applying the k-means algorithm. So, in the proposed anchor box regression layer, the fuzzy c-means is implemented in order to improve over the traditional k-means in distinctions between positive and negative anchor boxes, which will be illustrated in the experimental section below.

### 3.4. Classifier Network

During pulmonary nodule image detection, a considerable number of false positive detections frequently arise. In order to reduce or eliminate these false positives, a careful design of the classification network is crucial. As depicted in Figure 1 above, the ROI layer feeds all detected targets to the three-dimensional classifier model, which then assesses whether each target is a malignant nodule.

In terms of classifier network selection, this study evaluated and compared the performance of four models: ResNet, DenseNet, MobileNet, and MixNet.

#### 3.4.1. ResNet

ResNet, also known as residual neural network, is a model architecture proposed by Kaiming He et al. in 2015 [20]. It aims to solve the disappearing gradient problem, which is when the number of network layers deepens, the overall performance does not increase, but network degradation causes the gradient to disappear. Many papers and experimental results show that using ResNet can train deeper neural networks without being affected by network degradation and, at the same time, achieve better performance.

The residual block serves as the fundamental unit in ResNet and plays a crucial role in effectively addressing the problem of network degradation; it is a variation of the residual block. While a typical residual block comprises two 3 × 3 convolution layers, the bottleneck block begins by reducing the dimension, proceeds with feature extraction, and then restores the dimension. This approach helps prevent a significant increase in the number of parameters, which can be a concern in deep neural network design.

The ResNet architecture is characterized by different numbers of layers, such as 18, 34, 50, 101, 152, and so on. The 18 and 34-layer versions are considered shallower neural networks, primarily composed of residual blocks. On the other hand, models with more than 50 layers use bottleneck blocks to mitigate the issue of parameter size increase associated with deep networks. In this study, ResNet-50 is chosen as the classifier.

#### 3.4.2. DenseNet

DenseNet, introduced by Gao Huang et al. in 2016 [21], is another deep neural network architecture. Its inspiration comes from the connection method used in ResNet’s residual blocks but takes it a step further by employing a more tightly connected block structure called dense connections. Traditional convolutional neural networks (CNNs) usually comprise convolutional and pooling layers, where each layer’s output is connected only to the previous layer. This design can limit the network’s ability to fully utilize features from previous layers during later stages of training, potentially leading to issues like network degradation and gradient vanishing. In a manner similar to ResNet, DenseNet utilizes densely connected blocks to repeatedly use previous layer features for training in each layer and passes these features backward, enhancing feature diversity.

The dense block serves as the fundamental building block of DenseNet. It consists of multiple convolutional layers organized in a sequence. Within each dense block, the input features pass through a set of convolutional layers. Importantly, the output of each layer is connected to the outputs of all previous layers, forming a dense connection structure. This means that each layer within a dense block receives input from all the preceding layers. This architecture allows features to be transmitted more freely throughout the network, enhancing the model’s performance.

DenseNet comes in various model architectures with different numbers of layers, including 121, 169, 201, 264 layers, and more. In this study, DenseNet-121 was chosen as the classifier.

#### 3.4.3. MobileNet

MobileNet, introduced by Andrew Howard et al. in 2017 [22], was designed with the goal of minimizing computational requirements during training while retaining effective detection capabilities. It is particularly well-suited for deployment on mobile and embedded devices.

MobileNet adopts depthwise separable convolution as an alternative to traditional convolution. Standard convolution involves the weighted summation of input channels, leading to a substantial increase in computation and parameters over several operations. To reduce computational costs, MobileNet decomposes standard convolution into two distinct steps: depthwise convolution and pointwise convolution. This separation helps optimize efficiency.

Depthwise convolution involves applying a distinct filter to each input channel, which allows for the exploration of channel correlations. Following this, pointwise convolution reduces the dimensionality of the output channels generated by depth convolution. This is achieved through the application of a 1 × 1 convolution kernel for pointwise linear combinations.

#### 3.4.4. MixNet

MixNet, introduced by Mingxing Tan et al. [23], focuses on enhancing model performance and efficiency. Like MobileNet, MixNet employs a combination of depthwise separable convolution and standard convolution operations at varying scales. This approach allows for more comprehensive feature expression and results in improved overall performance.

Mixed convolution is a central component of MixNet. It aims to address the high computational burden associated with traditional convolution when processing various channels. MixConv blends convolution kernels of different scales, such as 1 × 1, 3 × 3, 5 × 5, to conduct depth-separable convolution operations. This approach enables the model to retain a certain level of feature expression capability while minimizing computational requirements.

#### 3.4.5. Multi-Backbone Classifier

Since the classifiers investigated above are similar in depth, this investigation proposes a multi-backbone classifier that combines the best comparable performers based on the results of experiments, which are the ResNet and the DenseNet, and combines them in a multi-backbone classifier. The union of the generated anchor boxes is then combined with the anchor boxes proposed by the FCM-based regression layer.

#### 3.4.6. Attention Model

Attention models [24] have been traditionally applied to machine translation and have shown adaptability in various neural network architectures in various fields, such as natural language processing and computer vision. The primary function of the attention mechanism is to enhance the importance of crucial features while reducing the weight assigned to less important features, thereby mitigating their impact on the model. In order to enhance our model’s ability to distinguish between positive and negative samples, an attention model is incorporated into the classifier network, following a similar architecture presented by Li et al. [25]. In this investigation, two attention models are employed for performance comparison purposes.

A. Type I Coordinate Attention Model (CA-I)

**Figure 4:**
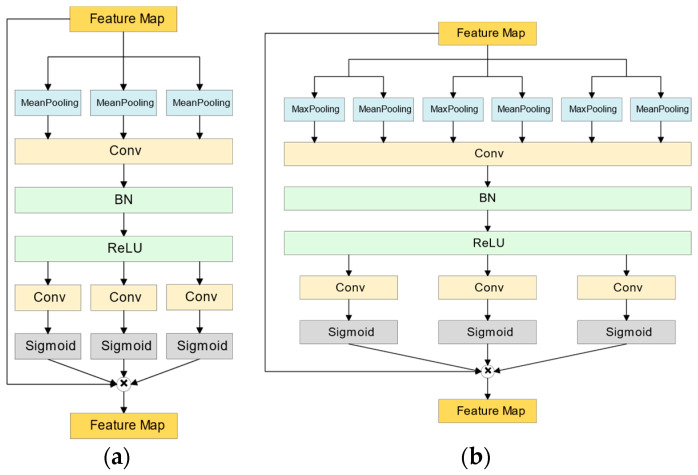
The structure diagrams of the models of coordinate attention networks proposed to be used in the classifier: (**a**) CA-I and (**b**) CA-II. Blocks of the same color perform similar functions.

The idea of Coordinate Attention (CA), introduced by Hou et al. [26], is an attention mechanism similar to SENet (squeeze-and-excitation networks) [27]. It conducts one-dimensional global average pooling on input features along various directions within each dimension, aggregating them to create multiple one-dimensional feature vectors. These vectors are then encoded into multiple attention maps, with each attention map capturing long-term dependencies along one spatial direction of the features. This makes coordinate attention particularly effective in making the model more sensitive to relevant spatial and directional information, thereby improving the precision in localizing and identifying target features. Once the information related to location is preserved in the newly generated attention maps, these maps are multiplied with the input features to yield weighted features. This adjustment allows the network to prioritize meaningful regions, thus further enhancing model performance. The structural diagram of CA-I is illustrated in Figure 4a. In this figure, BN is short Batch Normalization.

B. Type II Coordinate Attention model (CA-II)

In this section, we introduce a modified version of the coordinate attention architecture, denoted as the type II coordinate attention model (CA-II). To implement structural changes in the Coordinate Attention model, we drew inspiration from the CBAM (convolutional block attention module) method proposed by Woo et al. [28]. The adapted structure is depicted in Figure 4b. Unlike the SENet and coordinate attention models mentioned earlier, CBAM alters the pooling stage by combining global average and maximum pooling simultaneously. Subsequent processing is conducted after extracting features from both channels. Woo et al. explained that this combination allows the integration of spatial information and the capture of global average features with global average pooling, as well as the identification of salient features of specific targets with maximum pooling. This approach results in a richer feature representation. By globally assessing the features across different channels and determining the significance of each channel, the model can assign more attention to the most vital feature channels. In this study, we replaced the pooling process in the coordinate attention model with the pooling method from CBAM to evaluate its potential for achieving better results.

## 4. Results

In order to assess the effectiveness of the proposed RPN architecture, the multi-backbone classifier, fuzzy c-means-based regression, and the use of coordinate attention models, CA-I and CA-II, in the classifier layer, two experiments were conducted, and the performances were evaluated using various indicators.

### 4.1. Performance Indicators

To evaluate the system’s performance, this study employs several commonly used performance indicators [29]:

1. Accuracy: accuracy represents the proportion of correct predictions made by the model as expressed in Equation (2). Higher accuracy indicates the model’s ability to effectively distinguish between positive and negative samples. However, in cases where the proportion of negative samples is imbalanced, high accuracy may not reflect the overall performance of the model.
2. Precision: precision is the probability that targets classified as positive are actually positive, as expressed in Equation (3). A higher precision indicates a lower probability of false-positive results, reducing the chances of misdiagnosis.
3. Sensitivity: sensitivity, also known as recall, measures the proportion of actual positive samples that are correctly classified as positive, as expressed in Equation (4). Higher sensitivity implies a stronger ability to identify positive samples and is suitable for tasks with a potential for a large number of false-positive targets.
4. Specificity: specificity assesses the model’s ability to correctly identify negative samples, reducing the likelihood of false-positive results. The formula is expressed in Equation (5).

These indicators help evaluate the model’s performance and its ability to correctly classify both positive and negative samples in the context of medical image analysis.

(2) $$\text{Accuracy}=\frac{\mathrm{TP}+\mathrm{TN}}{\mathrm{TP}+\mathrm{FP}+\mathrm{TN}+\mathrm{FN}}$$

(3) $$\text{precision}=\frac{\mathrm{TP}}{\mathrm{TP}+\mathrm{FP}}$$

(4) $$\text{Sensitivity}=\frac{\mathrm{TP}}{\mathrm{TP}+\mathrm{FN}}$$

(5) $$\text{Specificity}=\frac{\mathrm{TN}}{\mathrm{FP}+\mathrm{TN}}$$

The F1 score is used as an indicator to evaluate the performance of the model. It combines the harmonic average of the precision and recall rates, considering both the accuracy and completeness of the classifier for a comprehensive evaluation of the model’s performance. The F1 score is calculated as follows:

(6) $$F1\;\mathrm{score}=\frac{2\;\times \;\mathrm{precision}\;\times \;\mathrm{recall}\;}{\mathrm{precision}+\mathrm{recall}\;}$$

### 4.2. Experiment 1

**Figure 5:**
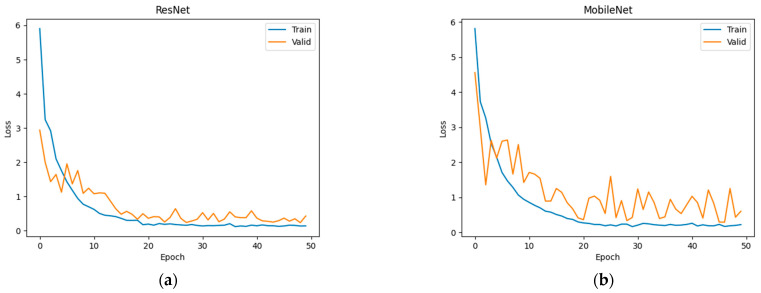

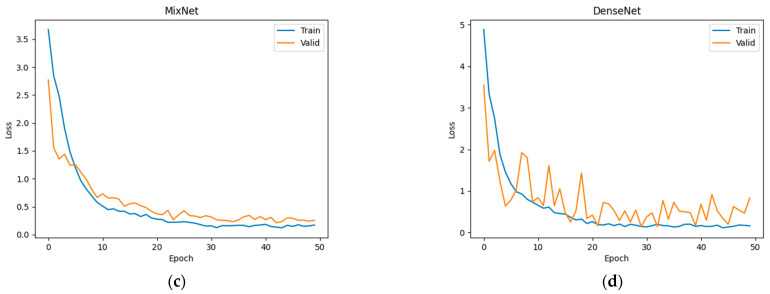
The loss curves during training (blue) and validation (orange) of (**a**) ResNet, (**b**) MobileNet, (**c**) MixNet, and (**d**) DenseNet in combination with fuzzy c-means clustering.

In the experiment, the region proposal network (RPN) generates anchors using either the k-means or fuzzy c-means clustering methods with varying cluster numbers. The pulmonary nodule detection is then performed using four different classifier networks in a 5-fold cross-validation method. The results are presented below. Figure 5 shows the loss curves of training and validation of several combinations of deep learning networks in combination with fuzzy c-means clustering.

A. Classifier using ResNet-50

In this experiment, both k-means and fuzzy c-means clustering methods with different cluster numbers (K) were employed. Table 1 and Table 2 indicate that the fuzzy c-means method exhibits superior accuracy, sensitivity, and specificity when the number of clusters is set to 5. Consequently, in the following parts of the study, the fuzzy c-means method with a cluster number of K = 5 is used due to its superior performance in these metrics. The highest value in each column is marked in red. The purpose of this experiment is to compare the effectiveness of the traditional k-means vs. FCM.

B. Classifier using DenseNet-121

In this part of the experiment, both k-means and fuzzy c-means clustering methods with different cluster numbers (K) were applied. Table 3 and Table 4 clearly demonstrate that the fuzzy c-means method outperforms k-means in terms of accuracy, sensitivity, and specificity when the number of clusters is set to 5. Notably, fuzzy c-means achieved the highest sensitivity of 0.9582, which is the best result in Experiment 1.

C. Classifier using MobileNet V2

In this part of the experiment, k-means and fuzzy c-means clustering methods with different cluster numbers (K) were applied, and the results are presented in Table 5 and Table 6. MobileNet, being a relatively lightweight neural network, exhibited lower overall performance compared to other networks. For example, when the number of clusters was set to 5, the highest sensitivity achieved using fuzzy c-means clustering was only 0.937.

D. Classifier using MixNet

In this part of the experiment, both k-means and fuzzy c-means clustering with different cluster numbers (K) were applied. The results are summarized in Table 7 and Table 8. When the number of clusters was set to 5, the model using FCM achieved a sensitivity of 0.9456 and an accuracy of 0.9381, which was slightly better than the model using k-means. Overall, the performance is not as strong as ResNet or DenseNet but is comparable to lightweight networks like MobileNet.

### 4.3. Experiment 2

In Experiment 1, we evaluated four classifier networks with both k-means and fuzzy c-means clustering methods for anchor generation. Based on the results from Table 1 to Table 8, it was evident that using ResNet and DenseNet with the fuzzy c-means clustering method when the number of clusters is set to 5 yielded the best results.

In Experiment 2, we used this configuration as a constant condition and added the attention mechanism, as described in Section 3.4.6, to observe its effects on the overall network performance. The results are presented in Table 9 and Table 10. It was observed that using either ResNet or DenseNet with coordinate attention I (CA-I) achieved an overall accuracy of 95.32%. However, the performance with CA-II falls below expectations. ResNet has higher accuracy and specificity, while DenseNet has higher sensitivity and F1 score. Using other attention models may be beneficial for classification but may result in slightly lower classification performance on certain indicators compared to not using an attention model at all.

## 5. Discussion

The goal of this investigation is to determine whether simple changes to the components in the region proposal network (RPN) can have incremental, but consistent improvements in the task of the locating the areas in lung CT scans that contain malignant tumors. The results of the investigation shows that our proposal modification to the RPN does achieve consistent, though incremental, improvements.

The results from Experiment 1 showed that ResNet and DenseNet outperformed MobileNet and MixNet. Additionally, using the fuzzy c-means method for anchor generation in RPN, along with either ResNet or DenseNet, led to improved overall performances. Moreover, the addition of the CA-I attention model contributed to increased accuracy and sensitivity. To provide further context, this approach utilized a hybrid 2D and 3D faster R-CNN model architecture for detection. A comparison was made with previous methods that also employed a 2D hybrid 3D or full 3D model architecture, and the results are presented in Table 11, with the highest value in each column marked in red. So, they are the most likely candidates for building a multi-backbone classifier.

The architectural model comparisons revealed interesting insights. The full 3D method developed by Liu et al. achieved an accuracy of 0.961. While the sensitivity achieved by Shuvo’s method was similar to that of Liu et al., the accuracy was slightly lower, which might be due to sample proportion issues affecting feature extraction and training.

In comparison to Liu et al.’s method, this approach demonstrated a more balanced trade-off between sensitivity and accuracy. When compared to the 2D plus 3D architectural model, Nguyen et al. achieved a sensitivity of 0.938, along with accuracy and specificity values of 0.957 and 0.976, respectively. These results indicated a reduced likelihood of false positives and misclassifications, contributing to an overall accuracy improvement. On the other hand, the method used in this study achieved a sensitivity as high as 0.975 when not using the method of Banu et al.

Considering Banu et al.’s approach, which used PAWE/CAWE, it was expected to have better performance in distinguishing positive and negative samples. However, it exhibited poor sensitivity in identifying positive samples, with a value of only 0.917, resulting in reduced accuracy. In contrast, the method employed in this study achieved a higher sensitivity of 0.962, which surpassed the methods of both Banu et al. and Nguyen et al.

In general, there may not always be a clear-cut performance advantage between 2D plus 3D and all-3D models. However, when the performances are comparable, 2D plus 3D models tend to have fewer parameters than their all-3D counterparts. This difference in model complexity can impact factors such as processing time and computational cost. As such, designers should consider these factors when choosing between the two architectural approaches.

## 6. Conclusions

Our proposed approach introduces a pulmonary nodule detection model with a hybrid architecture that combines a 2D feature extraction network to capture detailed features with a 3D CNN classifier. In comparison to pure 2D CNN methods, this approach considers more spatial information, leading to improved accuracy and sensitivity. Additionally, when compared to pure 3D CNN methods, this hybrid model significantly reduces computational demands during feature extraction, making the training process more efficient while maintaining high accuracy. While promising results have been achieved, shown in Table 11, in that our proposed method achieved the highest sensitivity while lagging only slightly in terms of accuracy and specificity respective to previously published methods, so there is a need to continue working to enhance the methods and architectures and continue to improve the overall model performance.

## Figures and Tables

**Table 1:** Performance indicators using ResNet and k-means clustering detection.

	Index	Accuracy	Precision	Sensitivity	Specificity	F1 Score
Cluster						
K = 3		0.9287	0.9146	0.9414	0.9167	0.9278
K = 5		0.9389	0.9265	0.9498	0.9286	0.9380
K = 7		0.9430	0.9342	0.9498	0.9365	0.9419
K = 9		0.9409	0.9339	0.9456	0.9365	0.9397

**Table 2:** Performance indicators using ResNet and FCM clustering detection.

	Index	Accuracy	Precision	Sensitivity	Specificity	F1 Score
Cluster						
K = 3		0.9348	0.9259	0.9414	0.9286	0.9336
K = 5		0.9491	0.9421	0.9540	0.9444	0.9480
K = 7		0.9450	0.9380	0.9498	0.9405	0.9439
K = 9		0.9389	0.9336	0.9414	0.9365	0.9375

**Table 3:** Performance indicators using DenseNet and k-means clustering detection.

	Index	Accuracy	Precision	Sensitivity	Specificity	F1 Score
Cluster						
K = 3		0.9145	0.8988	0.9289	0.9008	0.9136
K = 5		0.9430	0.9342	0.9498	0.9365	0.9419
K = 7		0.9369	0.9298	0.9414	0.9325	0.9356
K = 9		0.9348	0.9259	0.9414	0.9286	0.9336

**Table 4:** Performance indicators using DenseNet and FCM clustering detection.

	Index	Accuracy	Precision	Sensitivity	Specificity	F1 Score
Cluster						
K = 3		0.9287	0.9180	0.9372	0.9206	0.9275
K = 5		0.9491	0.9385	0.9582	0.9405	0.9482
K = 7		0.9409	0.9339	0.9456	0.9365	0.9397
K = 9		0.9328	0.9256	0.9372	0.9286	0.9314

**Table 5:** Performance indicators using MobileNet and k-means clustering detection.

	Index	Accuracy	Precision	Sensitivity	Specificity	F1 Score
Cluster						
K = 3		0.9104	0.8980	0.9205	0.9008	0.9091
K = 5		0.9226	0.9069	0.9272	0.9087	0.9218
K = 7		0.9226	0.9136	0.9289	0.9167	0.9212
K = 9		0.9206	0.9098	0.9289	0.9127	0.9193

**Table 6:** Performance indicators using MobileNet and FCM clustering detection.

	Index	Accuracy	Precision	Sensitivity	Specificity	F1 Score
Cluster						
K = 3		0.9435	0.9020	0.9247	0.9524	0.9132
K = 5		0.9300	0.8582	0.9372	0.9266	0.8960
K = 7		0.9300	0.8610	0.9331	0.9286	0.8956
K = 9		0.9246	0.8533	0.9247	0.9246	0.8876

**Table 7:** Performance indicators using MixNet and k-means clustering detection.

	Index	Accuracy	Precision	Sensitivity	Specificity	F1 Score
Cluster						
K = 3		0.9206	0.9132	0.9247	0.9167	0.9189
K = 5		0.9369	0.9298	0.9414	0.9325	0.9356
K = 7		0.9348	0.9295	0.9372	0.9325	0.9333
K = 9		0.9308	0.9218	0.9372	0.9246	0.9295

**Table 8:** Performance indicators using MixNet and FCM clustering detection.

	Index	Accuracy	Precision	Sensitivity	Specificity	F1 Score
Cluster						
K = 3		0.9246	0.8479	0.9331	0.9206	0.8884
K = 5		0.9381	0.8726	0.9456	0.9345	0.9076
K = 7		0.9367	0.8721	0.9414	0.9345	0.9054
K = 9		0.9314	0.8615	0.9372	0.9286	0.8978

**Table 9:** Performance indicators using ResNet with SENet, CA-I, and CA-II.

Attention Model	Accuracy	Precision	Sensitivity	Specificity	F1 Score
-	0.9491	0.9421	0.9540	0.9444	0.9480
SENet	0.9511	0.9461	0.9540	0.9484	0.9500
CA-I	0.9532	0.9463	0.9582	0.9484	0.9522
CA-II	0.9470	0.9419	0.9498	0.9444	0.9458

**Table 10:** Performance indicators using DenseNet with SENet, CA-I, and CA-II.

Attention Model	Accuracy	Precision	Sensitivity	Specificity	F1 Score
-	0.9491	0.9385	0.9582	0.9405	0.9482
SENet	0.9409	0.9268	0.9540	0.9286	0.9402
CA-I	0.9532	0.9426	0.9623	0.9444	0.9524
CA-II	0.9450	0.9344	0.9540	0.9365	0.9441

**Table 11:** Performance comparison with previous methods.

Method	Architecture	Accuracy	Sensitivity	Specificity
Shuvo [9]	3D	0.936	0.931	n/a
Liu [12]	3D	0.961	0.933	n/a
Banu [14]	2D + 3D	0.913	0.917	0.935
Nguyen [15]	2D + 3D	0.957	0.938	0.976
Ours	2D + 3D	0.953	0.962	0.954

## Data Availability

No new data were generated or analyzed in support of this research.
